# *Lactobacillus plantarum* CCFM1143 Alleviates Chronic Diarrhea *via* Inflammation Regulation and Gut Microbiota Modulation: A Double-Blind, Randomized, Placebo-Controlled Study

**DOI:** 10.3389/fimmu.2021.746585

**Published:** 2021-10-15

**Authors:** Bo Yang, Yue Yue, Yang Chen, Mengfan Ding, Bowen Li, Linlin Wang, Qun Wang, Catherine Stanton, R. Paul Ross, Jianxin Zhao, Hao Zhang, Wei Chen

**Affiliations:** ^1^ State Key Laboratory of Food Science and Technology, Jiangnan University, Wuxi, China; ^2^ School of Food Science and Technology, Jiangnan University, Wuxi, China; ^3^ International Joint Research Laboratory for Pharmabiotics & Antibiotic Resistance, Jiangnan University, Wuxi, China; ^4^ Yancheng Tinghu District People’s Hospital, Yancheng, Jiangsu, China; ^5^ APC Microbiome Ireland, University College Cork, Cork, Ireland; ^6^ Teagasc Food Research Centre, Moorepark, Fermoy, Ireland; ^7^ National Engineering Research Center for Functional Food, Jiangnan University, Wuxi, China; ^8^ Wuxi Translational Medicine Research Center, Jiangsu Translational Medicine Research Institute Wuxi Branch, Wuxi, China

**Keywords:** probiotics, *Lactobacillus plantarum*, chronic diarrhea, gut microbiota, clinical trial

## Abstract

Irritable bowel syndrome with diarrhea and functional diarrhea are both functional bowel disorders that cause chronic diarrhea. Chronic diarrhea is closely related to daily life and the psychological condition of diarrhea in patients, and probiotics can play a significant role in alleviating chronic diarrhea in some research. Lactobaccilus plantarum CCFM1143 can relieve diarrhea in mice caused by enterotoxigenic Escherichia coli (ETEC); however, its clinical effects remain unclear. This study aimed to assess the effects of CCFM1143 as a therapy for chronic diarrhea patients. Fifty-five patients with chronic diarrhea were randomly assigned into the probiotic group (n = 28) and the placebo group (n = 27), receiving the routine regimen with or without probiotics for 4 weeks, respectively. CCFM1143 can mitigate the apparent clinical symptoms and improve the health status and quality of life of patients. In addition, it could inhibit the increase in interleukin 6 (IL-6) and the decrease in motilin; modulate the short-chain fatty acids, especially acetic and propionic acids; and regulate the gut microbiota, particularly reducing the abundance of Bacteroides and Eggerthella and enriching the abundance of Akkermansia, Anaerostipes, and Terrisporobacter. In addition, treatment with probiotics showed clinical effectiveness in managing chronic diarrhea when compared with the placebo group. The findings could help to develop and further the application of probiotics for chronic diarrhea.

## Introduction

Chronic diarrhea is a complex and common problem faced by primary care clinicians. Its prevalence worldwide is estimated to be 3%–20% (1). It also has a significant negative impact on the health-related quality of life, causes high healthcare utilization, and increases the economic burden (2). Generally, chronic diarrhea lasts longer than 28 days, and in addition to the duration of symptoms, it tends to occur without a clear onset (1). Stool frequency (more than two bowel movements per day) and/or stool consistency (loose or watery stools) are the common criteria to define diarrhea (3). Recent literature has recommended using abnormal stool consistency rather than stool frequency, as it correlates best with the objective measures of whole gut transit time (4).

Irritable bowel syndrome with diarrhea (IBS-D) and functional diarrhea (FD) are the most common causes of chronic diarrhea in western populations (5) and are attributed to functional bowel disorder. Previous studies have combined the incidences of IBS-D and functional diarrhea to report the prevalence of chronic diarrhea in the general population (6), but the underlying pathophysiology remains poorly understood. According to Roman standards, the main difference in the symptoms between FD and IBS-D is the presence of abdominal pain (4). It is particularly challenging when diarrhea becomes chronic and therefore less likely to resolve spontaneously.

IBS-D and FD are common diseases accompanied by disturbances in the intestinal function. Some studies have reported that probiotics could improve IBS symptoms in IBS-D patients, which include *Lactobacillus brevis* KB290 and β-carotene (7) and a combination of *Lactobacillus acidophilus* CL1285, *Lactobacillus casei* LBC80R, and *Lactobacillus rhamnosus* CLR2 (8). In addition, *Bifidobacterium infantis* Y1 and *Bifidobacterium breve* Y8 ameliorated the clinical symptoms and changed the diversity and composition of the gut microbiota in chronic diarrhea patients (9). Probiotics, including *Lactobacillus* and *Bifidobacterium*, were favored over placebo for their effects on the overall symptoms of IBS (10, 11). However, some studies have reported contrasting effects, which may be related to strain-specific properties and a change in the host itself (12). Since the prevalence and clinical picture of IBS show great variations between race and ethnicity, differences in the gut microbiota between individuals might also exert a role in IBS (13). Although imbalance in the gut microbiota is a possible mechanism driving the development of IBS, a lot of previous research focused on the clinical outcomes. Thus, extensive clinical studies are still required to elucidate the role the gut microbiota play in the development of chronic diarrhea and to design specialized therapy for patients. Previous studies have shown that *Lactobacillus plantarum* CCFM1143 can relieve diarrhea caused by enterotoxigenic *Escherichia coli* (ETEC) (14). In this study, chronic diarrhea patients were assigned into a probiotic group and a placebo group to evaluate the efficacy of *L. plantarum* CCFM1143 in relieving chronic diarrhea, which will provide not only new evidence for probiotics as an alternative therapy to alleviate chronic diarrhea but also a foundation of knowledge for exploring the mechanism of probiotic treatment in alleviating chronic diarrhea.

## Materials and Methods

### Subjects

**Figure 1:**
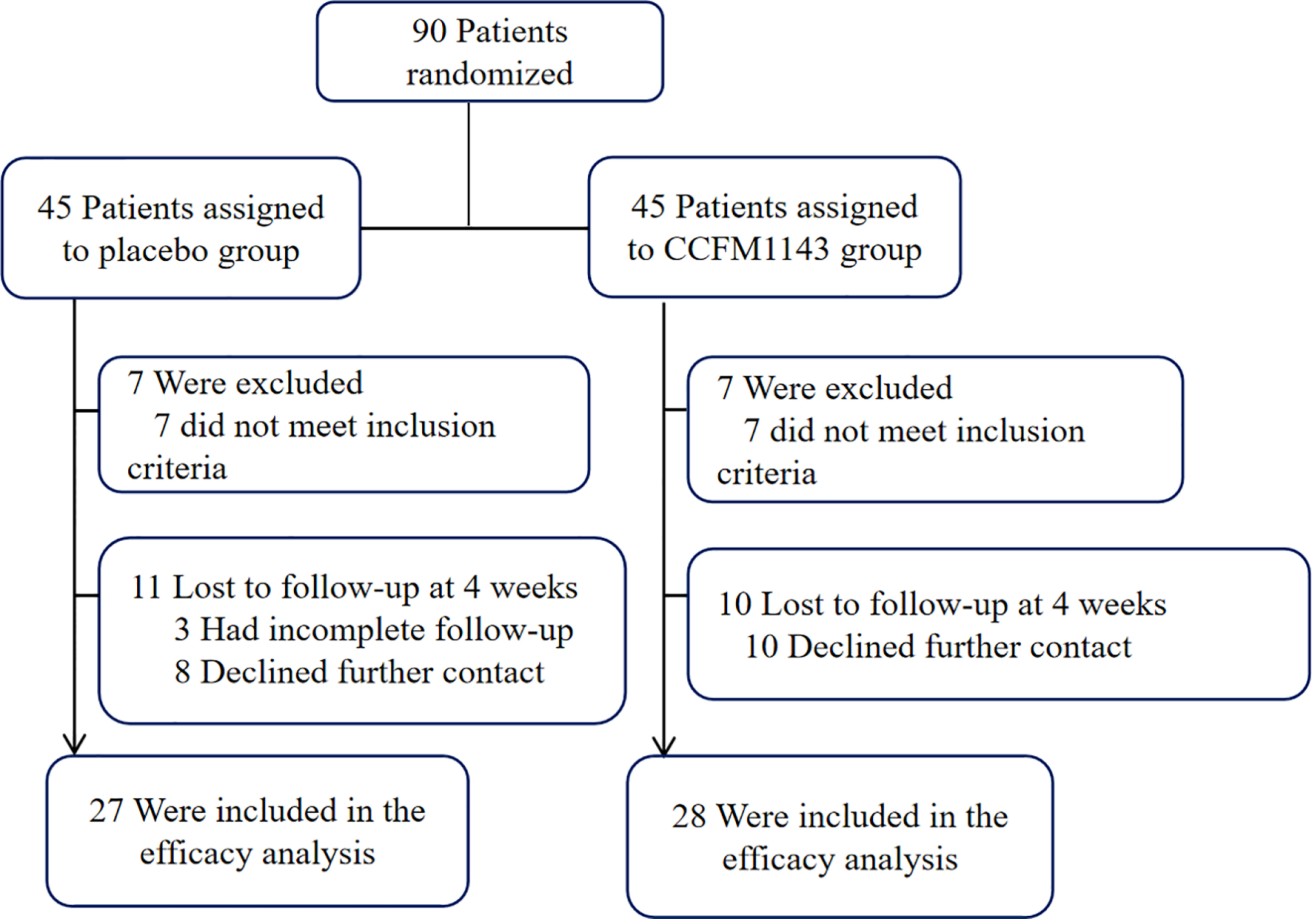
Clinical course of *Lactobacillus plantarum* CCFM1143 relieving chronic diarrhea.

Ninety patients were randomly assigned to the probiotic and placebo groups (**Figure 1**). The inclusion criteria for patients with diarrhea were as follows: 1) males and females 18–65 years old; 2) Rome IV standard was used as the diagnostic criterion. The frequency of defecation is greater than or equal to three times/day. According to the Bristol Stool Scale, normal stool of types 3 and 4 are used as a reference. Types 5, 6, and 7 can be regarded as diarrhea, as shown in **Table 1**. 3) Not using any drugs that can affect gastrointestinal motility in the past weeks; 4) the subjects or their guardians have signed an informed consent and have good compliance; and 5) routine blood, urine, and stool tests before treatment and liver and kidney function tests are all within the normal range. The exclusion criteria were pregnancy, drug abuse, alcohol addiction, and any immunodeficiency or organic disease. Participants who had taken medications within 4 weeks prior to this trial were excluded.

**Table 1:** Bristol stool scale.

Bristol typing	Form	Description
1. Nut-like stool	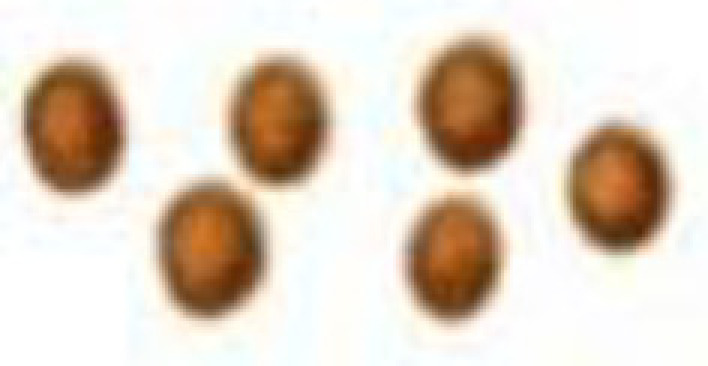	Hard, small pieces, like rabbit dung
2. Dry hard stool	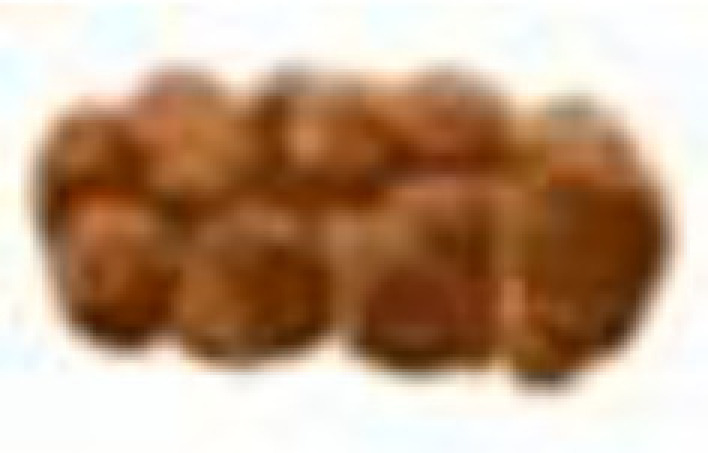	The texture is hard, with multiple small pieces stuck together, in the shape of a sausage.
3. Wrinkled stool	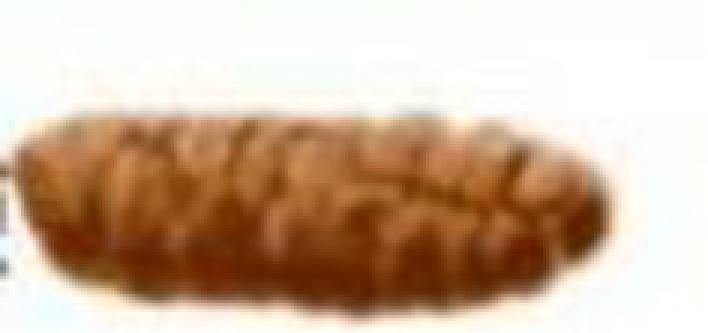	The surface is covered with cracks and is sausage-like.
4. Banana-shaped stool	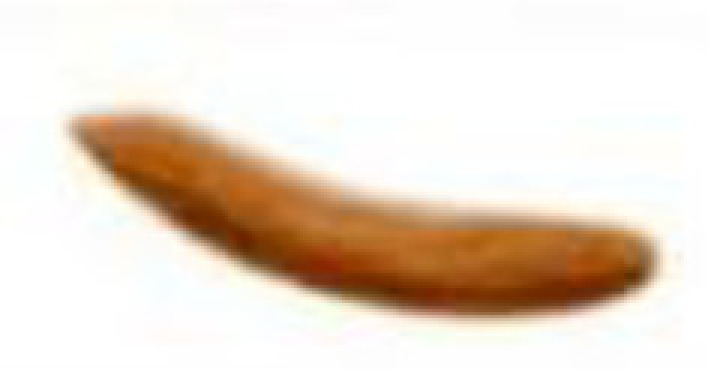	Soft texture, smooth surface, sausage-like
5. Soft stool	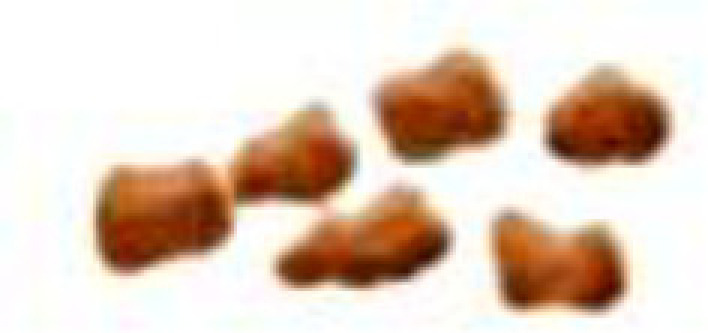	Soft semi-solid with uneven edges
6. Slightly shaped stool	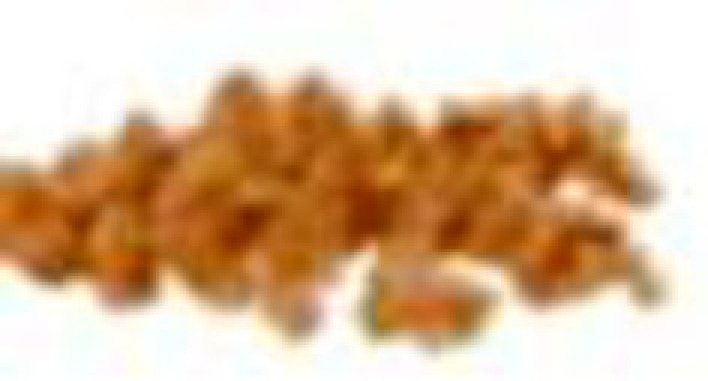	Soft flakes, porridge with rough edges or no fixed shape
7. Watery stool	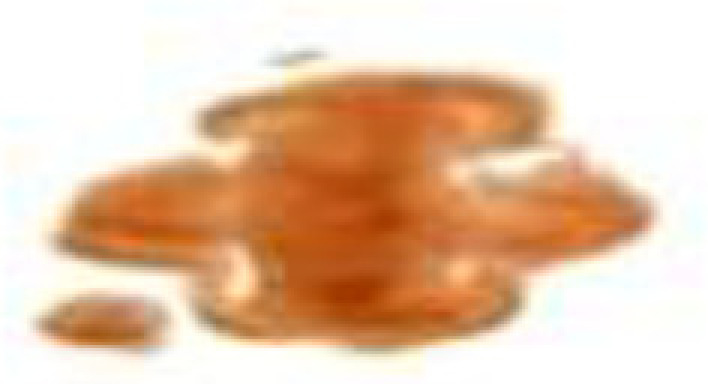	Watery, completely liquid without solids

### Clinical Trial Design

During the study period, volunteers need to record the consumption of probiotic preparations and emergency medicines, as well as information related to defecation, and record the frequency of diarrhea, stool consistency (Bristol Stool Form Scale, BSFS), pain, and abdominal distension. Patients with diarrhea were divided into two large groups, namely, the *L. plantarum* CCFM1143 intervention group [3.52 × 10^9^ colony forming units (CFU)/day] and the placebo (maltodextrin) group. Probiotics can be taken directly, mixed with warm water (not exceeding 37°C) or mixed with fresh milk (yogurt or lactic acid bacteria products are not recommended), and taken 30 min after meals, once a day, for a period of 30 days. Stool collection was carried out in the fifth week and related indicators were tested. The experimental preparation period was 1 week and the experimental process was 4 weeks, with a total duration of 5 weeks. The patients were recruited through the Yancheng Tinghu District People’s Hospital (Yancheng, Jiangsu Province, China). The trial was approved by the Ethnic Committee of the hospital (no. ET2020088).

### Assessment of Clinical Outcomes

Patients with diarrhea underwent routine physical examinations at the first visit and in the fourth week of follow-up visits. The characteristics examined included height, weight, blood pressure, blood glucose, blood lipids, four blood routines, urine routine, stool routine, and liver function. The defecation frequency and stool status were also recorded to evaluate the relieving effect. At the same time, abdominal symptoms [we refer to the Irritable Bowel Syndrome Severity Scoring System (IBS-SSS) for the evaluation of IBS severity] and daily life points [we refer to the diarrhea section of the Irritable Bowel Syndrome Quality of Life (IBS-QOL) table) were scored (15, 16). Stool satisfaction, the 36-Item Short Form Health Survey (SF-36) diarrhea mood score, and the SF-36 overall health score were calculated (17). A lower score means an improvement in the quality of life of patients with diarrhea.

### Neurobiological Factors, Cytokines, and MTL Evaluation

Serum was provided by The Tinghu People’s Hospital, Yancheng, and the commercialized kits purchased from Wuhan Elabscience Biotechnology Co., Ltd (Wuhan, China) were used to determine tumor necrosis factor-α (TNF-α), interleukin-6 (IL-6), motilin (MTL), and vasoactive intestine peptide (VIP) and 5-hydroxytryptamine (5-HT) following the manufacturer’s instructions.

### Determination of SCFAs

Briefly, short-chain fatty acids (SCFAs) were extracted using a previous method, with some modifications (18). A known mass of feces (50–100 mg) was weighed directly into sterile tubes, followed by soaking in 500 μl of saturated NaCl solution for 30 min. The soaked matter was homogenized and acidified with 40 μl of 10% sulfuric acid. One milliliter of ether was added to the acidified homogenates and vortexed before centrifugation at 18,000 × *g* at 4°C for 15 min. Of the supernatants, 500 μl was carefully filtered through a 0.22-μm pore filter and transferred into a gas phase vial for gas chromatography–mass spectrometry analysis.

### High-Throughput Sequencing of the Gut Microbiota

Metagenomic DNA from the fecal samples was obtained using a FastDNA Spin Kit for Feces (cat. no. 6570200, MP Biomedicals, Irvine, CA, USA) according to the manufacturer’s instructions. The V3–V4 region of the 16S rRNA gene was PCR amplified from microbial genomic DNA (forward primer, 5′-AYT GGG YDT AAA GNG-3′; reverse primer, 5′-TAC NVG GGT ATC TAA TCC-3′) as described previously (19). The sequencing data obtained from the Illumina MiSeq PE300 platform was processed using QIIME 2 (20). Then, Marker Data Profiling (https://www.microbiomeanalyst.ca/) was used for alpha diversity (Chao1 and Shannon indexes) and beta diversity analyses, in which analysis of the beta diversity was based on non-metric multidimensional scaling (NMDS) analysis. Linear discriminant analysis effect size (LEfSe) was performed online (http://huttenhower.sph.harvard.edu/galaxy).

### Statistical Analysis

Data were processed with GraphPad Prism and SPSS and displayed as the mean ± SEM of each group. The Mann–Whitney *U* test was used to analyze statistical significance. A *p* < 0.05 indicates a statistically significant difference.

## Results

### *L. plantarum* CCFM1143 Improved the Apparent Symptoms in Chronic Diarrhea Patients

The basic characteristics of patients with chronic diarrhea are shown in **Table 2**. The body mass index (BMI) values of the two groups were mostly concentrated in the range of normal (18.5–22.9 kg/m^2^) and overweight (23.0–27.9 kg/m^2^), which were roughly the same.

**Table 2:** Basic characteristics of patients with diarrhea.

Group	Male-to-female ratio	Age (years)	Height (cm)	Weight (kg)	BMI (kg/m^2^)		
					Normal	Overweight	Obese
Placebo	10:17	47.37 ± 12.77	165.15 ± 6.53	64.48 ± 9.86	12	14	1
*L. plantarum* CCFM1143	8:20	52.68 ± 9.1	163.58 ± 6.74	64.23 ± 10.8	14	12	2

There was no significant difference in the blood pressure, blood routine, serum biochemistry, and urine routine (baseline) of the enrolled diarrhea patients in the placebo and *L. plantarum* CCFM1143 groups (before intervention, Placebo-B, CCFM1143-B; after intervention, placebo-A, CCFM1143-A). The routine occult blood test of stool was negative and the microscopic examination was normal, indicating that there was no serious inflammatory disease. After the 4-week intervention, there was no significant difference between the placebo and *L. plantarum* CCFM1143 groups in those indicators. *L. plantarum* CCFM1143 showed no effects on the blood pressure, blood routine, serum biochemistry, and urine routine of patients with diarrhea (**Table 3**).

**Table 3:** Information on the routine physical examination for patients with diarrhea.

Item		Placebo-B	Placebo-A	CCFM1143-B	CCFM1143-A
Blood pressure	Systolic	118.75 ± 12.03	115.46 ± 10.57	120.46 ± 11.89	120.2 ± 8.82
	Diastolic	75.74 ± 9.69	75.35 ± 9.17	76.75 ± 8.71	76.2 ± 7.89
Blood routine	Red blood cell	4.68 ± 0.5	4.52 ± 0.47	4.51 ± 0.35	4.52 ± 0.29
	Mean corpuscular hemoglobin concentration	136.93 ± 23.89	135.31 ± 15.79	137.96 ± 11.88	139.54 ± 12.17
	Platelet	190.48 ± 47.32	202.04 ± 40.89	236.29 ± 51.48	224.46 ± 59.28
	White blood cell	5.88 ± 1.35	5.71 ± 1.16	5.84 ± 1.34	5.39 ± 1.12
Serum biochemistry	Alkaline phosphate (Alp)	67.38 ± 19.88	68.42 ± 21.55	62.54 ± 13.48	71.82 ± 14.95
	Alanine aminotransferase (Alt)	32.62 ± 20.62	23.54 ± 12.9	22.64 ± 9.73	29.88 ± 17.86
	Aspartate aminotransferase (Ast)	24.52 ± 6.42	22.38 ± 6.12	25.15 ± 14.29	25.79 ± 13.14
	Total bilirubin (Tbil)	17.31 ± 6.68	14.47 ± 4.52	15.84 ± 5.32	15.83 ± 5.83
	Urea (Bun)	5.05 ± 1.37	4.86 ± 1.01	5.75 ± 1.18	5.38 ± 1.23
	Creatinine (Cr)	68.59 ± 12.72	68.79 ± 16.38	60.05 ± 11.64	59.28 ± 12.4
	Uric acid (Ua)	352.78 ± 108.91	321.04 ± 79.59	299.42 ± 60.55	291.57 ± 65.11
	Glucose (Glu)	5.82 ± 1.55	5.84 ± 1.59	5.28 ± 1	5.54 ± 1.62
	Total cholesterol (Cho)	4.43 ± 0.73	4.54 ± 1.09	5.2 ± 0.99	5.2 ± 1.09
	Triglyceride (Tg)	1.7 ± 0.89	1.89 ± 1.19	1.33 ± 0.45	1.63 ± 0.73
	High-density lipoprotein cholesterol (hdl)	1.16 ± 0.24	1.13 ± 0.19	1.44 ± 0.19	1.42 ± 0.29
	Low-density lipoprotein (ldl)	2.59 ± 0.71	2.72 ± 0.79	3.15 ± 0.85	3.03 ± 0.71
	High-sensitivity C-reactive protein	3.47 ± 2.93	3.91 ± 2.71	2.8 ± 1.87	3.33 ± 3.69
Urine routine	Urine sugar	Negative	Negative	Negative	Negative
	Urine bilirubin (bil)	Negative	Negative	Negative	Negative
	Ketones (ket)	Negative	Negative	Negative	Negative
	Proportion (sg)	1.02 ± 0.003	1.02 ± 0.002	1.02 ± 0.01	1.02 ± 0.01
	pH value (pH)	6.08 ± 0.41	6.1 ± 0.57	5.76 ± 0.69	6.08 ± 0.91
	Urine protein (pro)	Negative	Negative	Negative	Negative
	Urobilinogen (ubg)	Negative	Negative	Negative	Negative
	Nitrite (nit)	Negative	Negative	Negative	Negative
	Leukocyte esterase (leu)	Negative	Negative	Negative	Negative
	Urine color	Yellow	Yellow	Light yellow	Yellow
	Clarity	Transparent	Transparent	Transparent	Transparent
Stool routine	Occult blood	Negative	Negative	Negative	Negative
	Microscopic examination of white blood cells	No abnormality	No abnormality	No abnormality	No abnormality
	Microscopic examination of red blood cells	No abnormality	No abnormality	No abnormality	No abnormality

Analysis of the visual indicators in patients with diarrhea showed that there was no significant difference between the placebo group and the *L. plantarum* CCFM1143 group at baseline (0 week) in bowel frequency and stool consistency (Bristol score) (**Table 4**). After the 4-week intervention, the placebo group had no improvement in defecation frequency and stool consistency, while the defecation frequency and Bristol score of patients in the *L. plantarum* CCFM1143 group have been significantly reduced, indicating that the chronic diarrhea symptoms had been partially relieved. The abdominal symptom score, daily life score, SF-36 diarrhea mood score, and SF-36 diarrhea overall health score of the placebo group and the CCFM1143-treated group showed no significant difference before and after intervention. Moreover, reductions of the abdominal symptom score, daily life score, and SF-36 diarrhea overall health score were higher in the *L. plantarum* CCFM1143 group than those of the placebo group, but were not significant.

**Table 4:** Relief scores of *L. plantarum* CCFM1143.

Index	Placebo				*L. plantarum* CCFM1143			
	0 week	4 weeks	Effect	*p*	0 week	4 weeks	Effect	*p*
Bowel frequency	3.48 ± 0.51	3.22 ± 0.51	−0.26	0.073	3.25 ± 0.57	2.93 ± 0.53	−0.32	0.044
Stool consistency	5.60 ± 0.89	5.26 ± 1.19	−0.34	0.758	5.68 ± 0.66	5.28 ± 0.84	−0.40	0.041
Abdominal symptom score	4.15 ± 1.66	4.29 ± 1.59	0.14	0.953	5.18 ± 2.22	4.54 ± 2.15	−0.64	0.350
Daily life score	31.93 ± 10.76	33.00 ± 12.73	1.07	0.946	37.14 ± 14.97	32.93 ± 15.18	−4.21	0.266
Stool satisfaction	2.41 ± 0.79	2.29 ± 0.67	−0.12	0.664	2.96 ± 0.94	2.50 ± 1.02	−0.46	0.113
SF-36 diarrhea mood score	13.70 ± 2.83	16.23 ± 4.92	2.53	0.384	14.64 ± 2.78	14.04 ± 2.92	−0.60	0.764
SF-36 diarrhea overall health score	10.33 ± 2.86	10.70 ± 2.45	0.37	0.927	12.14 ± 1.73	11.29 ± 2.52	−0.85	0.601

### Effect of *L. plantarum* CCFM1143 on Neurobiological Factors, Cytokines, and MTL in Chronic Diarrhea Patients

**Figure 2:**
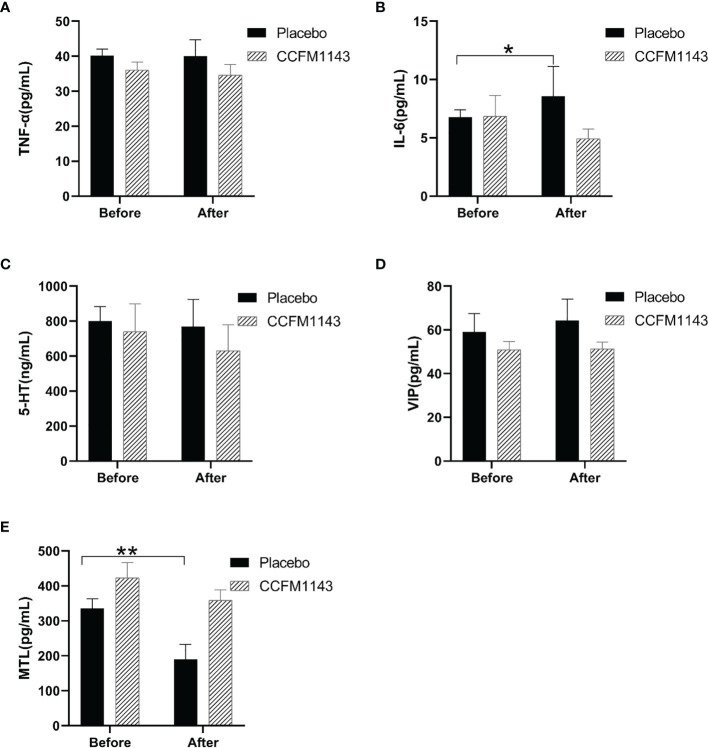
Effect of *Lactobacillus plantarum* CCFM1143 on serum neurobiological factors, cytokines, and motilin (MTL) in patients with diarrhea. **(A–E)** Concentrations of tumor necrosis factor alpha (TNF-α) **(A)**, interleukin-6 (IL-6) **(B)**, 5-hydroxytryptamine (5-HT) **(C)**, vasoactive intestine peptide (VIP) **(D)**, and MTL **(E)**. *p < 0.05, **p < 0.01.

To further explore the effect of *L. plantarum* CCFM1143 on the immune response, intestinal motility, and nerve-related indicators of patients with diarrhea, human serum TNF-α, IL-6, MTL, 5-HT, and VIP were measured. The results showed that, at baseline (before intervention), there was no significant difference in TNF-α, IL-6, MTL, 5-HT, and VIP between the placebo and *L. plantarum* CCFM1143 groups (**Figure 2**). After 4 weeks of intervention, in the placebo group, IL-6 was significantly increased (*p* < 0.05), the MTL level was significantly decreased, VIP had a rising trend but was not significant, and TNF-α and 5-HT were not modified. However, after the intervention with *L. plantarum* CCFM1143, TNF-α, IL-6, MTL, and 5-HT all showed a tendency to decrease and had a certain inhibitory effect, although there was no significant difference, and VIP was affected at a certain extent before and after the intervention (**Figure 2**). Although *L. plantarum* CCFM1143 had no significant effect on MTL regulation, placebo treatment significantly reduced the MTL level, which also reflected the effectiveness of *L. plantarum* CCFM1143 on MTL regulation (**Figure 2**).

### Effect of *L. plantarum* CCFM1143 on Fecal SCFAs in Chronic Diarrhea Patients

**Figure 3:**
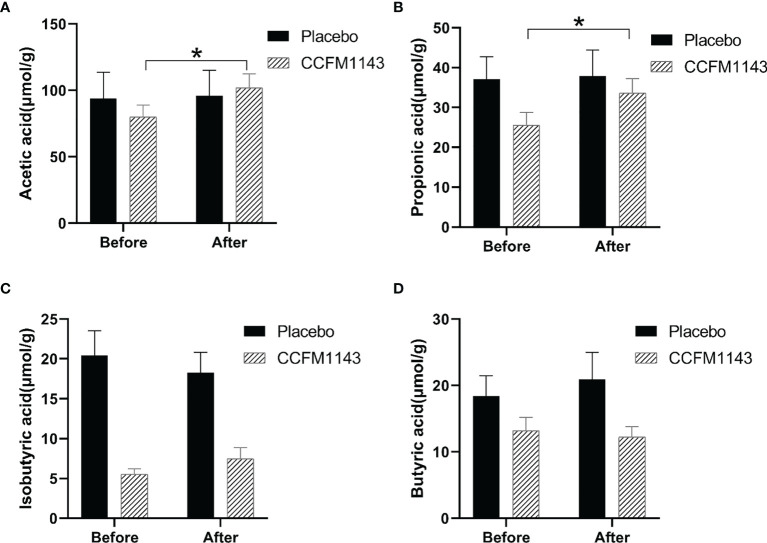
Effect of *Lactobacillus plantarum* CCFM1143 on short-chain fatty acids (SCFAs) in patients with diarrhea. **(A–D)** Concentrations of acetic acid **(A)**, propionic acid **(B)**, isobutyric acid **(C)**, and butyric acid **(D)** in feces. *p < 0.05.

In order to explore the effect of *L. plantarum* CCFM11143 on SCFAs in chronic diarrhea patients, GC-MS analysis was performed on human stool samples. After 4 weeks of intervention, there were no significant changes in acetic acid, propionic acid, isobutyric acid, and butyric acid in the placebo group. It is worth noting that *L. plantarum* CCFM1143 had a significant increase in the contents of acetic acid and propionic acid, but had no significant effect on isobutyric acid and butyric acid (**Figure 3**).

### Modulation of *L. plantarum* CCFM1143 on Gut Microbiota in Chronic Diarrhea Patients

**Figure 4:**
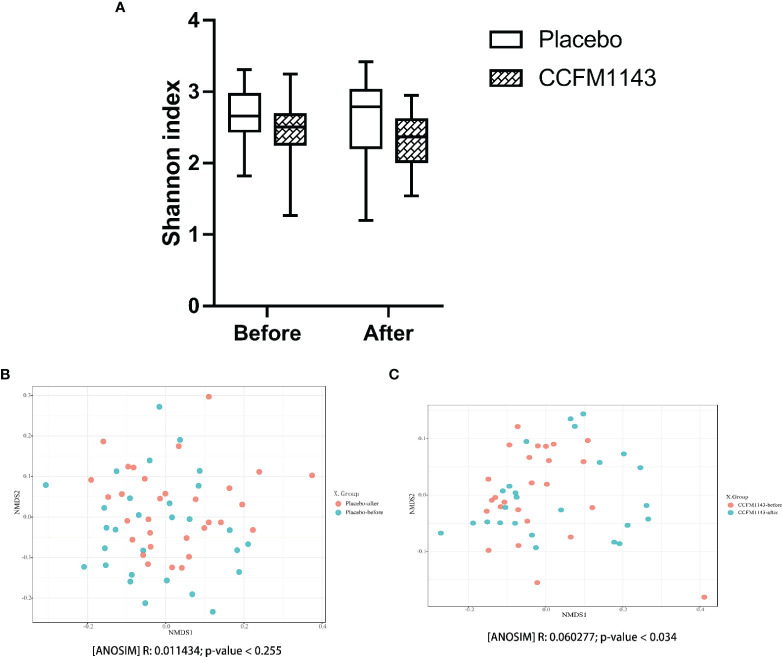
Effect of *Lactobacillus plantarum* CCFM1143 on the diversity of the gut microbiota in patients with diarrhea. **(A)** Alpha diversity indicated by the Shannon index. **(B)** Beta diversity of the placebo group indicated by non-metric multidimensional scaling (NMDS). **(C)** Beta diversity of the *L. plantarum* CCFM1143 group indicated by NMDS.

In order to evaluate the influence of *L. plantarum* CCFM1143 on the diversity of the gut microbiota in chronic diarrhea patients, the Chao1 and Shannon indexes were used to evaluate the alpha diversity and NMDS analysis was used to analyze the beta diversity of the gut microbiota. Chronic diarrhea patients in the placebo and *L. plantarum* CCFM1143 groups did not show a significant change in the diversity of the gut microbiota before and after the intervention (**Figure 4A**). There was no significant change in the beta diversity of the gut microbiota in the placebo group before and after the intervention, while the beta diversity of the gut microbiota in the *L. plantarum* CCFM1143-treated patients before and after the intervention was changed significantly, indicating that after 4 weeks of continuous intervention, *L. plantarum* CCFM1143 changed the diversity of gut microbiota in chronic diarrhea patients (**Figures 4B, C**).

**Figure 5:**
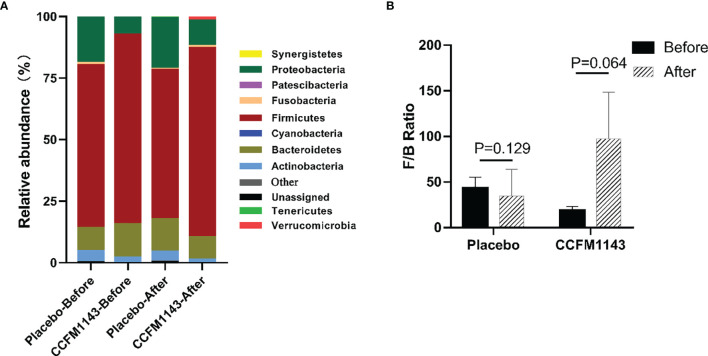
Effect of *Lactobacillus plantarum* CCFM1143 on the phylum level of the gut microbiota in patients with diarrhea. **(A)** Microbial distribution at the phylum level. **(B)** The Firmicutes and Bacteroidetes (F/B) ratio.

In order to further analyze the influence of *L. plantarum* CCFM1143 on the profiles of the gut microbiota in patients with chronic diarrhea, we examined the phylum abundance. The results showed that the four main phyla in the gut microbiota of patients with chronic diarrhea were Firmicutes, Bacteroidetes, Proteobacteria, and Actinomycota, among which Firmicutes had the highest proportion, while Proteobacteria had a higher relative abundance in chronic diarrhea patients than in healthy subjects (**Figure 5A**). The placebo group had no significant effect on these four phyla after the intervention, which was consistent with the placebo group having no change in the gut microbiota diversity after the intervention. Moreover, *L. plantarum* CCFM1143 treatment significantly reduced the abundance of Bacteroidetes. In addition, *L. plantarum* CCFM1143 had no significant effect on Proteobacteria and Actinomycetes. Moreover, the Firmicutes and Bacteroidetes (F/B) ratio in the CCFM1143 treatment showed an upward trend, while that in the placebo group showed no significant difference (**Figure 5B**).

**Figure 6:**
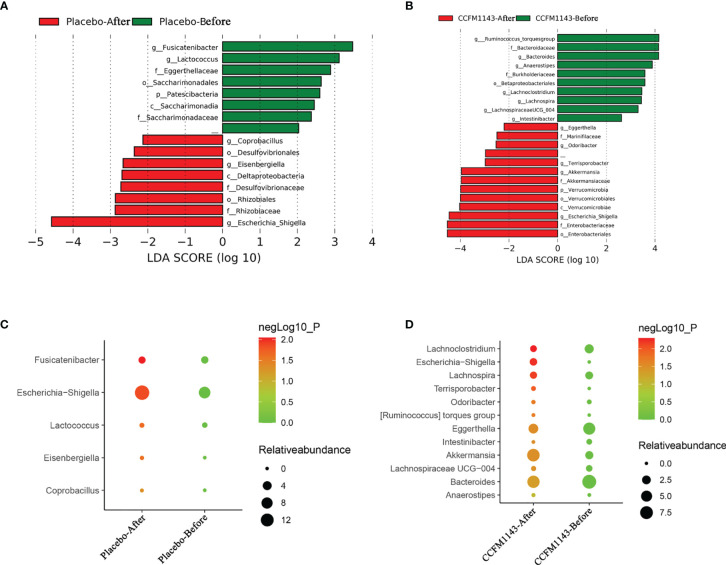
Effect of *Lactobacillus plantarum* CCFM1143 on the different genera of the gut microbiota in patients with diarrhea. **(A)** Distribution histogram of the placebo group based on linear discriminant analysis (LDA), with a log LDA score above 2.0. Significant taxa were labeled and annotated with *tags in the right panel*. **(B)** Distribution histogram of the *L. plantarum* CCFM1143 group based on LDA, with a log LDA score above 2.0. Significant taxa were labeled and annotated with *tags in the right panel*. **(C)** Relative abundance of the different microorganisms in the placebo group. **(D)** Relative abundance of the different microorganisms in the *L. plantarum* CCFM1143 group.

In order to further explore the influence of *L. plantarum* CCFM1143 on the changes of the intestinal genera in patients with chronic diarrhea, LEfSe was used to analyze the abundance of genera in order to determine the statistically significant biomarkers. The results showed that the different genera in the placebo group before intervention were *Fusicatenibacter* and *Lactococcus*, while those of the *L. plantarum* CCFM1143 group were the *Ruminococcus torques* group, *Bacteroides*, *Anaerostipes*, *Lachnoclostridium*, *Lachnospira*, *Lachnospiraceae* UCG-004, and *Intestinibacter*. After intervention, the different genera of the placebo group were *Coprobacillus*, *Eisenbergiella*, and *Escherichia–Shigella*, while the different genera in the *L. plantarum* CCFM1143-treated group were *Eggerthella*, *Odoribacter*, *Terrisporobacter*, *Akkermansia*, and *Escherichia–Shigella* (**Figures 6A, B**). Further analysis of the relative abundance of the different genera between the placebo and *L. plantarum* CCFM1143 groups showed that placebo treatment insignificantly decreased *Escherichia*–*Shigella*, and the adjustment changes to the other genera were small. However, *L. plantarum* CCFM1143 administration caused relatively rich variations of the different microbes, in which it significantly reduced the relative abundance of *Bacteroides*, *Eggerthella*, *Lachnoclostridium*, and *Lachnospira* and increased the relative abundance of *Akkermansia*, *Anaerostipes*, *Terrisporobacter*, and *Escherichia–Shigella*. Before and after the intervention, the relative abundance of *Escherichia–Shigella* in the placebo group increased by 7.24%, while that in the *L. plantarum* CCFM1143-treated group increased by 1.47%, which was a significant reduction compared with that in placebo treatment (**Figures 6C, D**).

## Discussion

IBS-D and FD are common chronic diarrhea diseases, and variations in the gut microbiota among individuals have been considered a possible cause (21). Probiotics have shown the potential to alleviate the clinical symptoms of chronic diarrhea (22, 23). The purpose of this study was to explore the relieving effect of *L. plantarum* CCFM1143 on the symptoms of chronic diarrhea. Thus, a randomized, placebo-controlled clinical trial was designed and performed, which showed effects of improvement in the quality of life and mental state, regulation of the immune responses, modulation of SCFAs, and alleviation of gut microbiota dysbiosis.

A BMI between 18.5 and 22.9 kg/m^2^ is considered normal, between 23.0 and 27.9 kg/m^2^ is the overweight range, and a BMI ≥28 kg/m^2^ denotes obesity (6). Before and after the intervention, the routine physical examination indicators were not changed significantly in chronic diarrhea patients. However, *L. plantarum* CCFM1143 treatment significantly decreased the defecation frequency and Bristol stool score. It was shown that the abdominal distension, defecation frequency, and stool abnormality rate of IBS-D patients treated with a multi-strain probiotic formulation were significantly lower than those before treatment (8), which was consistent with our current results. The mixture of *Lactobacillus* and *Bifidobacterium* could improve the quality of life of IBS-D patients (24). *Lactobacillus gasseri* significantly improved the diarrhea symptoms in patients with IBS-D, such as abdominal pain, bloating, daily routine, and average bowel frequency (25). Meanwhile, *B. infantis* M-63 effectively improved the symptoms, quality of life, and the SF-36 score in patients with IBS after 3 months of intervention. However, *L. plantarum* CCFM1143 treatment showed no significant effect on the quality of life and stool satisfaction. *L. plantarum* CCFM1143 played a certain role in improving the symptoms of diarrhea, and no adverse events occurred during the trial period and after the follow-up; hence, the use of *L. plantarum* CCFM1143 as a dietary intervention may be a safe and effective alternative strategy to relieve chronic diarrhea.

It is well known that inflammation in patients with diarrhea is high. Intervention with *Bifidobacterium* and *Lactobacillus* for 4 weeks in patients with atopic dermatitis did not significantly relieve the level of pro-inflammatory cytokines in the serum (26), and the results were similar to those of the current study, which may be related to the short probiotic intervention time and to uncontrollable factors such as patients. The significant increase in the level of IL-6 in the current placebo group indicated that *L. plantarum* CCFM1143 intervention had a better regulation on IL-6. A previous report showed that the serum IL-6 and TNF-α levels in patients with IBS-D were significantly lower after taking *Bifidobacterium longum* ES1 compared with those before treatment (27), but it should be noted that these patients took *B. longum* ES1 for 8 or 12 weeks. Therefore, the reduction of IL-6 in patients with chronic diarrhea by *L. plantarum* CCFM1143 treatment was not significant, which may be related to the number of viable *Lactobacillus* taken and the duration of treatment. MTL is an excitatory gastrointestinal hormone with a main physiological function of affecting gastrointestinal motility (28). In the study, administration of *L. plantarum* CCFM1143 inhibited the decrease of MTL compared with the placebo group. *Bifidobacterium* trifecta viable capsules could significantly improve the gastrointestinal hormones in patients with IBS-constipation (IBS-C) and alleviate their clinical symptoms (29). 5-HT is an important neurotransmitter in the brain–gut axis and is one of the key mediators of intestinal motility, secretion, and sensation and can also induce high sensitivity of the visceral afferent nerve and the intestinal nervous system, resulting in abdominal pain, abdominal distension, and other symptoms. 5-HT has always been the subject of interest in the evaluation of the pathophysiological mechanism of IBS (30). The level of 5-HT in patients with IBS-C was significantly lower than that before probiotics intervention (29). However, *L. plantarum* CCFM1143-treated patients showed insignificant decreases in the levels of 5-HT. VIP is distributed in the nervous system and the gastrointestinal tract and is involved in the regulation process of neurosecretion and relaxation of the gastrointestinal smooth muscle. In addition, VIP is an inhibitory gastrointestinal hormone that can directly cause lower esophageal sphincter (LES) relaxation, which is the main cause of gastroesophageal reflux disease (31). It has been reported that VIP is associated with watery diarrhea syndrome (32). Although *L. plantarum* CCFM1143 treatment showed no significant effect on MTL regulation, the MTL level in the placebo group was significantly reduced, which also reflected the effectiveness of *L. plantarum* CCFM1143 on MTL regulation. In general, *L. plantarum* CCFM1143 can help regulate the immune response and inhibit the increase of IL-6 and the decrease of MTL in patients with diarrhea, which has a certain correlation with the decrease of the diarrhea-related scores.

An earlier report showed that *L. casei* increased the contents of SCFAs when alleviating antibiotic-related diarrhea, and the intake of probiotics and dietary fiber would affect the composition of the gut microbiota, thereby promoting the utilization of SCFAs (33). *L. plantarum* CCFM1143 intervention significantly increased the contents of acetic acid and propionic acid. The increase of SCFAs such as propionic acid can contribute to the host immune response (34). SCFAs (particularly acetate, propionate, and butyrate) could alleviate TNF-α- or lipopolysaccharide-induced endothelial activation by inhibiting the production of pro-inflammatory cytokines (e.g., IL-6) (35). In summary, *L. plantarum* CCFM1143 can alleviate diarrhea symptoms and modulate inflammation mainly related to the production of SCFAs. This was similar to a previous result showing that *L. plantarum* CCFM1143 increased the contents of acetic acid and propionic acid in ETEC-infected diarrhea mice (14).

Probiotics could change the composition of the gut microbiota in patients with IBS-D or FD clinically. It has been found that changes of the gut microbiota may be related to the improvement of the clinical characteristics (9), which is somewhat similar to the current results. In general, the gut microbiota of healthy people is composed of four main bacterial phyla, namely, Firmicutes, Bacteroidetes, Actinomycetes, and Proteobacteria, of which Proteobacteria are usually less than 1%. In addition, it has been proven that Bacteroidetes is closely related to the occurrence of human diarrhea (36); the F/B ratio was often related to the occurrence of diseases. With a lower F/B ratio, the risk of disease occurrence was greater (37). In general, *L. plantarum* CCFM1143 treatment can significantly reduce the abundance of Bacteroidetes and increase the abundance of Firmicutes to restore the gut microbiota. A previous study that performed phylogenetic analysis showed that 16 butyric acid producers isolated from the cecum of chickens were associated with four different lineages in Firmicutes (38). It is known that exogenous harmful substances activated the NLRP3 inflammasomes through *Escherichia*–*Shigella*, thereby inducing lung tissue damage in broilers (39). Among them, *Escherichia* was known to increase the risk of pathogenic invasion and has the potential to result in severe invasive infections (40). *Bacteroides* has been proven to be the cause of diarrheal diseases (41); however, *Akkermansia* can reduce inflammation (42). For instance, *L. plantarum* CCFM8610 could increase the relative abundance of *Anaerostipes* when alleviating IBS-D, which has a strong butyric acid production capacity (43). *Terrisporobacter* played a probiotic role as a beneficial intestinal bacterium (44). These results suggested that the relief of diarrhea by *L. plantarum* CCFM1143 may be related to its regulation of the gut microbiota composition. The mixed bacterial powder of *L. casei* Zhang, *Bifidobacterium animalis* spp. *lactis* V9, and *L. plantarum* P-8 was reported to reduce the relative abundance of *Bacteroides*, *E. coli*, and *Citrobacter* in the intestine of patients with IBS and to increase the relative abundance of *Bifidobacterium* and *Butyricicoccus*. These gut microbiota changes had a relation with the improvement of the clinical symptoms of IBS (45). Patients with IBS-D consuming IgA-coated bacteria had a more significant increase in the relative abundance of *Escherichia*–*Shigella* (46). Therefore, it is worth noting that, similar to previous reports, *L. plantarum* CCFM1143 treatment increased the abundance of *Escherichia*–*Shigella*, which had a close relation with the complicated diet of patients.

## Concluding Remarks

Generally, the use of probiotics showed clinical effectiveness compared to placebo in managing chronic diarrhea. *L. plantarum* CCFM1143 can significantly alleviate the bowel frequency and Bristol stool score, inhibit the increase in IL-6 and the decrease in MTL, regulate the gut microbiota by reducing the abundance of harmful bacteria and increasing the abundance of beneficial bacteria, and modulate SCFAs. The current results could help further the development and application of functional probiotic products for chronic diarrhea.

## Data Availability Statement

The datasets presented in this study can be found in online repositories. The names of the repository/repositories and accession number(s) can be found below: https://www.ncbi.nlm.nih.gov/, PRJNA765509.

## Ethics Statement

The studies involving human participants were reviewed and approved by Ethics Committee of Tinghu District People’s Hospital, Yancheng, China. The patients/participants provided written informed consent to participate in this study.

## Author Contributions

BY, RR, and WC conceptualized the study. BY and YY helped with the methodology. MD and BL contributed to software development. BY and YY did the validation. LW performed formal analysis. BY, YY, and QW performed the investigation. HZ and WC helped with the resources. BY, YY, and HZ curated the data. BY, YC, and YY wrote the original draft. CS, RR, and WC reviewed and edited the manuscript. JZ helped with the visualization. RR, HZ, and WC supervised the study. JZ administered the project. WC acquired funding. All authors contributed to the article and approved the submitted version.

## Funding

This research was supported by the National Natural Science Foundation of China (32021005), National First-Class Discipline Program of Food Science and Technology (JUFSTR20180102), the Fundamental Research Funds for the Central Universities (JUSRP52003B), 111 Project (BP0719028), and Collaborative Innovation Center of Food Safety and Quality Control in Jiangsu Province.

## Conflict of Interest

The authors declare that the research was conducted in the absence of any commercial or financial relationships that could be construed as a potential conflict of interest.

## Publisher’s Note

All claims expressed in this article are solely those of the authors and do not necessarily represent those of their affiliated organizations, or those of the publisher, the editors and the reviewers. Any product that may be evaluated in this article, or claim that may be made by its manufacturer, is not guaranteed or endorsed by the publisher.

## References

[1] Schiller LR Pardi DS Sellin JH. Chronic Diarrhea: Diagnosis and Management. Clin Gastroenterol Hepatol (2017) 15:182–93. doi: 10.1016/j.cgh.2016.07.028 27496381

[2] Schiller LR. Evaluation of Chronic Diarrhea and Irritable Bowel Syndrome With Diarrhea in Adults in the Era of Precision Medicine. Am J Gastroenterol (2018) 113:660–9. doi: 10.1038/s41395-018-0032-9 29713027

[3] Mearin F Lacy BE Chang L Chey WD Lembo AJ Simren M. Bowel Disorders. Gastroenterology (2016) 150:1393–407. doi: 10.1053/j.gastro.2016.02.031 27144627

[4] Singh P Mitsuhashi S Ballou S Rangan V Sommers T Cheng V. Demographic and Dietary Associations of Chronic Diarrhea in a Representative Sample of Adults in the United States. Am J Gastroenterol (2018) 113:593–600. doi: 10.1038/ajg.2018.24 29610515

[5] Longstreth GF Thompson WG Chey WD Houghton LA Mearin F Spiller RC. Functional Bowel Disorders. Gastroenterology (2006) 130:1480–91. doi: 10.1053/j.gastro.2005.11.061 16678561

[6] Zhao YF Guo XJ Zhang ZS Ma XQ Wang R Yan XY. Epidemiology of Functional Diarrhea and Comparison With Diarrhea-Predominant Irritable Bowel Syndrome: A Population-Based Survey in China. PloS One (2012) 7:e43749. doi: 10.1371/journal.pone.0043749 22937091 PMC3427143

[7] Fuke N Aizawa K Suganuma H Takagi T Naito Y. Effect of Combined Consumption of *Lactobacillus Brevis* KB290 and β-Carotene on Minor Diarrhoea-Predominant Irritable Bowel Syndrome-Like Symptoms in Healthy Subjects: A Randomised, Double-Blind, Placebo-Controlled, Parallel-Group Trial. Int J Food Sci Nutr (2017) 68:973–86. doi: 10.1080/09637486.2017 28391736

[8] Preston K Krumian R Hattner J de Montigny D Stewart M Gaddam S. *Lactobacillus Acidophilus* CL1285, *Lactobacillus Casei* LBC80R and *Lactobacillus Rhamnosus* CLR2 Improve Quality-of-Life and IBS Symptoms: A Double-Blind, Randomised, Placebo-Controlled Study. Benef Microbes (2018) 9:697–706. doi: 10.3920/BM2017.0105 29888656

[9] Brigidi P Vitali B Swennen E Bazzocchi G Matteuzzi D. Effects of Probiotic Administration Upon the Composition and Enzymatic Activity of Human Fecal Microbiota in Patients With Irritable Bowel Syndrome or Functional Diarrhea. Res Microbiol (2001) 152:735–41. doi: 10.1016/s0923-2508(01)01254-2 11686387

[10] Sun J Kong C Qu X Deng C Lou Y Jia L. Efficacy and Safety of Probiotics in Irritable Bowel Syndrome: A Systematic Review and Meta-Analysis. Saudi J Gastroenterol (2020) 26:66–77. doi: 10.4103/sjg.SJG_384_19 31898645 PMC7279071

[11] Dale HF Rasmussen SH Asiller O Lied GA. Probiotics in Irritable Bowel Syndrome: An Up-to-Date Systematic Review. Nutrients (2019) 11:2048. doi: 10.3390/nu11092048 PMC6769995 31480656

[12] Whelan K. Probiotics and Prebiotics in the Management of Irritable Bowel Syndrome: A Review of Recent Clinical Trials and Systematic Reviews. Curr Opin Clin Nutr Metab Care (2011) 14:581–7. doi: 10.1097/MCO.0b013e32834b8082 21892075

[13] El-Salhy M Patcharatrakul T Hatlebakk JG Hausken T Gilja OH Gonlachanvit S. Chromogranin A Cell Density in the Large Intestine of Asian and European Patients With Irritable Bowel Syndrome. Scand J Gastroenterol (2017) 52:691–7. doi: 10.1080/00365521.2017.1305123 28346031

[14] Yue Y He Z Zhou Y Ross RP Stanton C Zhao J. *Lactobacillus Plantarum* Relieves Diarrhea Caused by Enterotoxin-Producing Escherichia Coli Through Inflammation Modulation and Gut Microbiota Regulation. Food Funct (2020) 11:10362–74. doi: 10.1039/d0fo02670k 33220669

[15] Francis CY Morris J Whorwell PJ. The Irritable Bowel Severity Scoring System: A Simple Method of Monitoring Irritable Bowel Syndrome and Its Progress. Aliment Pharmacol Ther (1997) 11:395–402. doi: 10.1046/j.1365-2036.1997.142318000.x 9146781

[16] Patrick DL Drossman DL Frederick IO Dicesare J Puder KL. Quality of Life in Persons With Irritable Bowel Syndrome: Development and Validation of a New Measure. Dig Dis Sci (1998) 43:400–11. doi: 10.1023/a:1018831127942 9512138

[17] Han B Shao Q Cong Y Guo S Mao X Wei R. Transcutaneous Electric Nerve Stimulation Over Acupoints for Patients With Diarrhea-Predominant Irritable Bowel Syndrome: Protocol for Systematic Review and Meta-Analysis. Med (Baltimore) (2018) 97:e13267. doi: 10.1097/MD.0000000000013267 PMC6320180 30572430

[18] Wang L Hu L Xu Q Jiang T Fang S Wang G. *Bifidobacteria* Exert Species-Specific Effects on Constipation in BALB/c Mice. Food Funct (2017) 8:3587–600. doi: 10.1039/c6fo01641c 28884754

[19] Tian P Wang G Zhao J Zhang H Chen W. *Bifidobacterium* With the Role of 5-Hydroxytryptophan Synthesis Regulation Alleviates the Symptom of Depression and Related Microbiota Dysbiosis. J Nutr Biochem (2019) 63:43–51. doi: 10.1016/j.jnutbio.2019.01.007 30743155

[20] Yan S Yang B Zhao J Stanton C Ross RP Zhang H. A Ropy Exopolysaccharide Producing Strain *Bifidobacterium Longum* Subsp. Longum YS108R Alleviates DSS-Induced Colitis by Maintenance of the Mucosal Barrier and Gut Microbiota Modulation. Food Funct (2019) 10:1595–608. doi: 10.1039/c9fo00014c 30806428

[21] Ringel Y. The Gut Microbiome in Irritable Bowel Syndrome and Other Functional Bowel Disorders. Gastroenterol Clin North Am (2017) 46:91–101. doi: 10.1016/j.gtc.2016.09.014 28164856

[22] Martoni CJ Srivastava S Leyer GJ. *Lactobacillus Acidophilus* DDS-1 and *Bifidobacterium Lactis* UABla-12 Improve Abdominal Pain Severity and Symptomology in Irritable Bowel Syndrome: Randomized Controlled Trial. Nutrients (2020) 12:363. doi: 10.3390/nu12020363 PMC7071206 32019158

[23] Ishaque SM Khosruzzaman SM Ahmed DS Sah MP. A Randomized Placebo-Controlled Clinical Trial of a Multi-Strain Probiotic Formulation (Bio-Kult^®^) in the Management of Diarrhea-Predominant Irritable Bowel Syndrome. BMC Gastroenterol (2018) 18:71. doi: 10.1186/s12876-018-0788-9 29801486 PMC5970461

[24] Cha BK Jung SM Choi CH Song ID Lee HW Joon Kim H. The Effect of a Multispecies Probiotic Mixture on the Symptoms and Fecal Microbiota in Diarrhea-Dominant Irritable Bowel Syndrome: A Randomized, Double-Blind, Placebo-Controlled Trial. J Clin Gastroenterol (2012) 46:220–7. doi: 10.1097/MCG.0b013e31823712b1 22157240

[25] Shin SP Choi YM Kim WH Hong SP Park JM Kim J. A Double Blind, Placebo-Controlled, Randomized Clinical Trial That Breast Milk Derived- *Lactobacillus Gasseri* BNR17 Mitigated Diarrhea-Dominant Irritable Bowel Syndrome. J Clin Biochem Nutr (2018) 62:179–86. doi: 10.3164/jcbn.17-73 PMC5874236 29610559

[26] Fang Z Lu W Zhao J Zhang H Qian L Wang Q. Probiotics Modulate the Gut Microbiota Composition and Immune Responses in Patients With Atopic Dermatitis: A Pilot Study. Eur J Nutr (2020) 59:2119–30. doi: 10.1007/s00394-019-02061-x 31342226

[27] Caviglia GP Tucci A Pellicano R Fagoonee S Rosso C Abate ML. Clinical Response and Changes of Cytokines and Zonulin Levels in Patients With Diarrhea Predominant Irritable Bowel Syndrome Treated With *Bifidobacterium Longum* ES1 for 8 or 12 Weeks: A Preliminary Report. J Clin Med (2020) 9:2353. doi: 10.3390/jcm9082353 PMC7464152 32717980

[28] Chu C Liaw Y. Hepatitis B Virus-Related Cirrhosis: Natural History and Treatment. Semin Liver Dis (2006) 26:142–52. doi: 10.1055/s-2006-939752 16673292

[29] Gao Z Ji J He X. Clinical and Experimental Study on Probiotics in the Treatment of Constipation Type Irritable Bowel Syndrome. Biomark Applic (2019) 3:138. doi: 10.29011/2576-9588.100038

[30] Harris LA Chang L. Alosetron: An Effective Treatment for Diarrhea-Predominant Irritable Bowel Syndrome. Womens Health (Lond) (2007) 3:15–27. doi: 10.2217/17455057.3.1.15 19803861

[31] Liang X Bi S Yang W Wang L Cui G Cui F. Evaluation of the Impact of Hepatitis B Vaccination Among Children Born During 1992-2005 in China. J Infect Dis (2009) 200:39–47. doi: 10.1086/599332 19469708

[32] Alm P Alumets J Håkanson R Owman O Sjöberg NO Sundler F. Origin and Distribution of VIP (Vasoac-Tive Intestinal Polypeptide)-Nerves in the Genitourinary Tract. Cell Tissue Res (1980) 205:337–47. doi: 10.1007/BF00232276 7357578

[33] Wong S Jamous A Driscoll JO Sekhar R Weldon M Yau CY. A *Lactobacillus Casei* Shirota Probiotic Drink Reduces Antibiotic-Associated Diarrhoea in Patients With Spinal Cord Injuries: A Randomised Con-Trolled Trial. Br J Nutr (2014) 111:672–8. doi: 10.1017/S0007114513002973 24044687

[34] Duscha A Gisevius B Hirschberg S Yissachar N Stangl GI Eilers E. Propionic Acid Shapes the Multiple Sclerosis Disease Course by an Immunomodu-Latory Mechanism. Cell (2020) 180:1067–80. doi: 10.1016/j.cell.2020.02.035 32160527

[35] Li M van Esch B Henricks PAJ Garssen J Folkerts G. Time and Concentration Dependent Effects of Short Chain Fatty Acids on Lipopolysaccharide- or Tumor Necrosis Factor α-Induced Endothelial Activation. Front Pharmacol (2018) 9:233. doi: 10.3389/fphar.2018.00233 29615908 PMC5867315

[36] Hahn AW Froerer C VanAlstine S Rathi N Bailey EB Stenehjem DD. Targeting *Bacteroides* in Stool Microbiome and Response to Treatment With First-Line VEGF Tyrosine Kinase Inhibitors in Metastatic Renal-Cell Carcinoma. Clin Genitourin Cancer (2018) 16:365–8. doi: 10.1016/j.clgc.2018.05.001 29858123

[37] Lynch SV Pedersen O. The Human Intestinal Microbiome in Health and Disease. N Engl J Med (2016) 375:2369–79. doi: 10.1056/NEJMra1600266 27974040

[38] Eeckhaut V Van Immerseel F Croubels S De Baere S Haesebrouck F Ducatelle R. Butyrate Production in Phylogenetically Diverse Firmicutes Isolated From the Chicken Caecum. Microb Biotechnol (2011) 4:503–12. doi: 10.1111/j.1751-7915.2010.00244.x PMC3815262 21375722

[39] Liu Q Zhou Y Li X Ma D Zhang M. Ammonia Induce Lung Tissue Injury in Broilers by Activating NLRP3 Inflammasome *via Escherichia/Shigella* . Poult Sci (2020) 99:3402–10. doi: 10.1016/j.psj.2020.03.019 PMC7597683 32616234

[40] Liderot K Ratcliffe P Lüthje P Thidholm E Özenci V. Microbiological Diagnosis of Eggerthella Lenta Blood Culture Isolates in a Swedish Tertiary Hospital: Rapid Identification and Antimicrobial Susceptibility Profile. Anaerobe (2016) 38:21–4. doi: 10.1016/j.anaerobe.2015.11.005 26612006

[41] Wick EC Sears CL. *Bacteroides* Spp. And Diarrhea. Curr Opin Infect Dis (2010) 23:470–4. doi: 10.1097/QCO.0b013e32833da1eb PMC3079340 20697287

[42] Shin NR Lee JC Lee HY Kim MS Whon TW Lee MS. An Increase in the *Akkermansia* Spp. Population Induced by Metformin Treatment Improves Glucose Homeostasis in Diet-Induced Obese Mice. Gut (2014) 63:727–35. doi: 10.1136/gutjnl-2012-303839 23804561

[43] Liu Y Yu X Yu L Tian F Chen W. *Lactobacillus Plantarum* CCFM8610 Alleviates Irritable Bowel Syndrome and Prevents Gut Microbiota Dysbiosis: A Randomized, Double-Blind, Placebo-Controlled, Pilot Clinical Trial. Engineering (2020) 7:376–85. doi: 10.1016/j.eng.2020.06.026

[44] Chen L Zhou W Zhou Y Tan T Du H Feng L. Analysis of the Effects of Nanosilver on Bacterial Community in the Intestinal Fluid of Silkworms Using High-Throughput Sequencing. Bull Entomol Res (2020) 110:309–20. doi: 10.1017/S0007485319000634 31559940

[45] Xu H Ma C Zhao F Chen P Liu Y Sun Z. Adjunctive Treatment With Probiotics Partially Alleviates Symptoms and Reduces Inflammation in Patients With Irritable Bowel Syndrome. Eur J Nutr (2020) 60:1–13. doi: 10.1007/s00394-020-02437-4 33225399

[46] Liu Y Yuan X Li L Lin L Zuo X Cong Y. Increased Ileal Immunoglobulin a Production and Immunoglobulin Acoated Bacteria in Diarrhea Predominant Irritable Bowel Syndrome. Clin Transl Gastroenterol (2020) 11:e00146. doi: 10.14309/ctg.0000000000000146 32352710 PMC7145038

